# Community volunteers can improve breastfeeding among children under six months of age in the Democratic Republic of Congo crisis

**DOI:** 10.1186/1746-4358-7-2

**Published:** 2012-02-24

**Authors:** Ghislain B Balaluka, Pépin S Nabugobe, Prudence N Mitangala, Nickel B Cobohwa, Carole Schirvel, Michèle W Dramaix, Philippe Donnen

**Affiliations:** 1 Centre de Recherche en Sciences Naturelles de Lwiro (CRSN), Bukavu, Democratic Republic of Congo; 2 School of Public Health, Université Libre de Bruxelles (ULB), Brussels, Belgium; 3 Centre Scientifique et Médical de l'Université Libre de Bruxelles pour ses activités de coopération (CEMUBAC), Brussels, Belgium

**Keywords:** Community volunteers, Exclusive breastfeeding, Growth, Malnutrition

## Abstract

**Background:**

Malnutrition is a major public health problem in developing countries and exclusive breastfeeding is an efficient strategy that can be used to prevent malnutrition and reduce child mortality. The objective of this study is to evaluate the effectiveness of community volunteers in promoting exclusive breastfeeding from birth in an area of endemic malnutrition.

**Methods:**

This evaluation analyzed the impact of the community-based nutrition project in Katana health district of the Democratic Republic of Congo from 2004 to 2006. Each of the villages in this sector had a nutritional village committee made up of five members responsible for continuously working to raise awareness of the importance of exclusive breastfeeding from birth among pregnant women and community leaders in their respective villages. The program worked with community volunteers with a mean age of 37 years, most of whom were married (86%). Eighty percent of the community volunteers had completed secondary school or a higher level of education. Data related to the period of exclusive breastfeeding and to the number of visits made to the health services for 208 children. The data were compared with data from 178 infants collected from another health sector, which had never developed a community-based nutrition program.

**Results:**

The duration of exclusive breastfeeding from birth (median, range) was 6 months (2 to 7) in the intervention area compared with 4 months (1 to 6) in the comparison area (p < 0.001). The proportion of infants receiving exclusive breastfeeding at six months of age was higher in the intervention area than in the comparison area: 57.7% (95% Confidence Interval, CI, 50.9 to 64.5) versus 2.7% (95%CI, 1.1 to 6.6) (p < 0.001). The intervention group had a higher mean weight at 12 months (standard deviation): 8.42 kg (1.41) compared to 7.97 kg (1.02), although this difference was not statistically significant (p = 0.055).

**Conclusions:**

The promotion of breastfeeding by community volunteers in an area of endemic malnutrition in rural Democratic Republic of Congo increased the duration of exclusive breastfeeding from birth.

## Background

During the first five years of this century, experts estimated the number of deaths recorded for children less than five years of age worldwide to be almost 11 million each year, of which the majority was in developing countries. According to these estimates 4 million deaths occurred in the 28 days following birth, and 35% to 53% of all deaths were associated with malnutrition [1-3]. The lack of intervention could jeopardize progress towards the United Nations' millennium development goals (MDG), which are to be achieved by 2015, in particular the fourth MDG, namely the reduction in mortality for children under the age of five [4].

In developing countries, millions of children under five years of age do not reach their physical and mental development potential because they are exposed to several risk factors (infections, malnutrition and micronutrient deficiencies), which compromise their development and against which it is possible to take action [5].

Exclusive breastfeeding (EBF) of a child during the first months of life is one of the strategies advocated to improve a child's chances of survival. Allen and Hector have shown the medical and economic benefits of this approach [6]. Jones et al. have demonstrated that if breastfeeding were carried out properly, the deaths of almost 1.3 million children per year could be averted [7]. Several studies have been conducted to determine the optimum length of time for exclusive breastfeeding and experts agree upon the need to promote exclusive breastfeeding until the age of six months and the continuation of breastfeeding until at least two years of age [8,9].

For all these reasons, the World Health Organization (WHO) and other United Nations organizations have sought to lead projects, which aim to encourage breastfeeding especially in developing countries, including exclusive breastfeeding until the age of 6 months [10]. This is an efficient strategy, which would be especially suited to developing countries where many households are not capable of paying the cost of substitute foods for infants. In developing countries, breastfeeding is more or less widespread for the majority of mothers. However, exclusive breastfeeding during the first six months is very low, as mothers introduce complementary foods early. In the Democratic Republic of Congo (DRC), the prevalence of EBF was estimated at 24% in 2002 [11] and 36% in 2006 [12]. In South Kivu province, the EBF prevalence was 21% in 2001 lower than that of the overall country [11].

Studies on promoting breastfeeding produce different results. A meta-analysis by Dyson et al. including seven studies on action taken to promote breastfeeding showed an improvement in breastfeeding practice in five studies [13]. A recent WHO publication looking at the results of projects and programs promoting breastfeeding shows that community projects can improve breastfeeding practices [14].

In the Democratic Republic of Congo's health system, health education and preparation for pregnant women to practice EBF during the first six months occurs during prenatal checkups. Once the women have given birth, they are encouraged at postnatal and "under-five" consultations to actually practice EBF during the first months. A policy document dealing with nutrition was published in 2000 by the Ministry of Public Health and made available to all the health districts. In this policy document, the health districts are called upon to develop a network of community healthcare workers and other organizations that are able to support the promotion of the exclusive breastfeeding strategy at the community level [15]. This health policy encourages using community volunteers or community healthcare workers to promote the EBF strategy in a manner similar to other public health programs [16,17]. We see it as necessary to focus on and further develop community strategies in order to extend the practice of exclusive breastfeeding to the age of six months.

The objective of this work is to study the effectiveness of community healthcare workers in promoting exclusive breastfeeding in the context of endemic malnutrition.

## Methods

### Study area

This study was carried out from 2004 to 2006 in the Katana and Walungu health districts located in the Province of South Kivu, in the eastern part of the DRC (between 1,500 and 2,000 meters in altitude). For over ten years this region had been politically unstable. The DRC is among those countries with a high infant mortality rate, the South Kivu region, where our study was carried out, having the highest in the country [11,12,18]. Malnutrition has been an endemic problem in the rural Kivu area for more than forty years [19,20].

In each health district we selected a health area which had a prevalence of acute malnutrition above 10% (Table 1). As was the case in the rest of the eastern part of the DRC, the selected regions had been unstable for more than ten years following an armed conflict, which broke out all throughout the region of the African Great Lakes. Ongoing refugee crises and population movements had been observed in episodes. This situation contributed to the weakness of the health indicators for children in the region [11,12].

**Table 1:** Characteristics of the two health areas (December 2003)

Indicator	Health area	
	Intervention area (Katana)	Comparison area (Walungu)
Total population	6,337	7,539
No. of health centers	1	1
No. of nutritional centers	1	1
Rate of global acute malnutrition (GAM)**	13.3%	12.0%
No. of children	208	178
Male children	51.9%	48.1%

**GAM = rate of acute global malnutrition calculated based on Weight for Height (WFH) ≤ -2Z-score and/or edema

### The health policy to promote breastfeeding in the DRC

In the DRC, raising awareness about the strategy of exclusive breastfeeding takes place when there is contact with the mother at antenatal clinics as part of the monitoring of a woman's pregnancy. For this occasion, the health center organizes awareness raising sessions around various themes for the benefit of mothers, and one of these sessions is about feeding the newborn baby. However, almost a third of pregnant women do not have access to antenatal clinic services in the country and the majority attend the clinic at the most once or twice during their final trimester [17]. After the birth of the baby, this raising of awareness continues through the "under-five" clinics. In the DRC health system, the "under-five" clinics are given the responsibility of evaluating the weight of an infant monthly, of immunization and to detect child health problems such as diarrhea, edema, pneumonia and prolonged fever. They also give health and nutritional advice.

### Intervention: Community-based nutrition program and subjects for the study

A community-based nutrition program had been developed in South Kivu since November 2003 by the Ministry of Public Health and the National Nutrition Program (PRONANUT). The provincial operational committee had selected the Lwiro health sector (Katana health district) to act as a pilot area when the program was implemented because a team with experience in setting up nutrition projects was already working in that sector. All the villages of the Lwiro health sector (Buhandahanda and Lwiro) were included in the program. Each of the villages selected was supplied with a team of five community healthcare workers trained in promoting good breastfeeding practices. These workers were then given the task of promoting exclusive breastfeeding from birth in their respective villages through door-to-door visits and community meetings. From 2004 to 2006, these community workers also took an active role in supervising the growth of infants by organizing community weighing sessions, which were supervised by members from the health district's central bureau. Weighing sessions were organized in each village on a monthly basis and each session was accompanied by a session to raise mothers' awareness of the benefits of breastfeeding and the need to practice exclusive breastfeeding from birth for a period of six months.

In the district of Walungu, used as the comparison area, no community-based nutrition project was being operated and there was no specific program to raise awareness about breastfeeding. The activities of the "under-five" clinics took place within the general health system. At that time, there were no community healthcare workers, and the male nurse in charge of the area was not informed that his health area had been selected as the control health area within the context of the study. Moreover, the health districts, Katana for "intervention" and Walungu for "comparison", are quite far apart and not adjoining. With regards to health indicators, these two districts are similar in regards to the prevalence of malnutrition, healthcare coverage and accessibility. These districts had also been used for a survey on breastfeeding carried out as part of the MICS2 survey in 2001. At that time and for all the households selected in the province, the prevalence of exclusive breastfeeding up to 6 months of age was 21% [11].

### Planning the study

This evaluation occurred following the implementation of a community-based nutrition program in the Lwiro health sector (Katana district) from 2004 to 2006. Pregnant women were identified at the start of their third trimester and were then given training by the community healthcare workers in their respective villages, preparing them to give birth and practice exclusive breastfeeding during the first six months of their newborn babies' lives. The infants were monitored from birth, for a period of one year.

### Community volunteers

In the intervention area, there already were community volunteers who took part in projects to supervise nutrition. Before the community volunteers were recruited, the population had been made aware of the problems of malnutrition in the community and the impact of this malnutrition on child mortality. Those promoting awareness voiced the need to set up local committees capable of supporting the population in its efforts to improve nutritional practices. After this initial work to raise awareness, the inhabitants of each village appointed five representatives who were to be part of the village committee based on the following criteria: personal motivation, proof of dedication to the community, integrity and good character, having a known job or occupation and being able to read and write. Priority was given to women so that there would be at least 60% on each committee.

The community volunteers selected were trained at the health district level about the key practices of community nutrition and about promoting breastfeeding. Key messages were defined so as to help the mothers understand the importance of exclusive breastfeeding from birth (Breast milk is the first food suitable for newborn babies and young infants; It protects the infant against diarrhea and other illnesses; It strengthens the immunity, without additional costs; Breastfeeding strengthens the psycho-emotional relationship between mother and child; Breastfeeding repeatedly stimulates breast milk production for the mother).

The community volunteers were organized into village committees and placed under the direct supervision of the health district team. In the DRC, the Health Policy encourages the use of community volunteers but they are not officially part of the health system and are not paid. The community volunteers in this study agreed to work for free. However, the health district agreed to call upon their services each time there would be health projects with the possibility of some remuneration (surveys, vitamin A supplementation campaign and polio immunization campaign).

In December 2007 we collected more information about the community volunteers from each village concerning their sex, age in years, civil status, level of study and profession:

• Civil status was recorded as married or living alone (unmarried, widower and divorced);

• Level of study (education) was categorized into three groups: primary studies only, secondary and technical studies, university studies;

• Profession was categorized into five groups: farmer; educator or employee; salesman; agriculturist or veterinarian; and other professions.

### Data collection and analysis

The following data were collected:

• Mother's name and address;

• Skilled assistance at birth: We considered a birth, which took place in the health center or maternity hospital, as a birth with "skilled assistance at delivery". Any birth that took place outside of health structures was considered as having occurred in an unsupervised environment;

• Weight at birth where this was available;

• Child's sex;

• Child's weight, measured every month during the "under-five" clinic session with Salter scales calibrated to within 100 grams accuracy. The child was undressed before being weighed. Children were generally weighed on Saturday mornings in the community chapels of the village by the same community volunteers;

• The diet the child was receiving: The mother was asked at each monthly visit if the child was receiving anything other than own mother's milk. An infant was considered as receiving exclusive breastfeeding from birth if he/she was fed since birth solely on own mother's milk without any other type of complement including liquid drinks such as sugar water or natural water. We considered only "exclusive breastfeeding" and not "predominant breastfeeding". Besides collecting data every month, the community volunteers organized house-calls every week to encourage the mothers to continue exclusive breastfeeding;

• After weighing the children, parents were made aware of the key practices of health: mainly the use of health services and hygiene (food and housing environment).

To estimate the effect of the project, we compared the development of these children with those coming from an area without community health workers during the same period. We selected the Walungu health district (Kalole) to serve as a basis for comparison (Table 1). The Walungu health district was chosen due to its resemblance to the Katana health district (demographic features, prevalence of malnutrition and rate of exclusive breastfeeding, social and economic characteristics, level of studies and profession of breastfeeding women, food availability and food habits, the rate of fertility and composition of households). In this context, more than 70% of the breastfeeding women were not schooled with only about 5% having completed primary education. The number of children per family was generally six with a mean inter-birth interval of two years. More than 80% of the women were farmers and lived under the poverty threshold. It was a typical African rural environment where the rate of exclusive breastfeeding was below 30% at six months [11,12,17-19].

The files from preschool consultation services were analyzed retrospectively but for the same period. Visits were organized to the homes of the selected children in order to confirm the age when feeding complements, including drinks, were introduced (the end of exclusive breastfeeding).

We registered a total of 224 children in the intervention area and 206 children in the comparison area for period lasting from 2004 to 2005. The analysis included 208 and 178 children respectively (after files were cleaned up and missing cases were excluded).

### Control and management of data

Data collection was supervised by a research team made up of one physician, three supervisory nutritionists and two registered nurses from the health centers.

Each village committee was supervised at least once every two months. At the end of the month, the health area village committee met with the senior nurse of the health center to validate reports from different villages before sending them to the health district office. Each month, meetings between the health district team and the research team were organized to validate reports made by community volunteers.

### Statistical methods

The data collected was entered into MS Access and analyzed using SPSS 12.0. The Mann- Whitney test was used to compare the medians for the length of exclusive breastfeeding and the number of weighings in each group. Pearson's Chi square test or Fischer's exact test were used to compare the proportion of children having EBF, stratified by age (1 to 6 months). We used the Student-t test to compare the mean of the weight at 3, 6, 9 and 12 months of follow-up in the two groups. We used Excel to produce the figure showing exclusive breastfeeding in both groups over time.

The protocol of this study was approved by the ethics committee of the Natural Sciences Research Center in Lwiro DRC (CRSN). As this research was planned as an evaluation of the community intervention, consent of individuals was not requested.

## Results

The demographic characteristics of the community volunteers are summarized in Table 2. There were slightly more women than men. The majority were married, less than 46 years old and had completed secondary school.

**Table 2:** Characteristics of community volunteers

Characteristic	Total		n	%
**Sex**	138	Women	81	58.7

		Men	57	41.3

**Mean age **[years (SD)]	138		36.7 (7.9)	

**Age group **(years)		20-35	66	47.8

		36-45	51	37.0

		46 years and up +	21	15.2

**Civil status**	138	Married	119	86.2

		Living alone	19	13.8

**Education**	136	Primary studies	27	19.9

		Secondary studies	105	77.2

		University studies	4	2.9

**Profession**	131	Farmer	66	50.4

		Educator/employee	40	30.5

		Salesman	15	11.5

		Agriculturist/veterinarian	4	3.1

		Other	6	4.6

The number of births with skilled assistance and the number of pre-school consultations (PSC) during the first year in both areas are presented in Table 3. The proportion of deliveries assisted by medical staff was significantly higher in the intervention area than in the comparison area as was the number of PSC sessions during the first year.

**Table 3:** Health performance - skilled assistance at delivery, PSC consultations

Variable	Intervention area	Comparison area	p value
Institutional deliveries or skilled assistance at delivery (%)	92.8	38.3	< 0.001

No. of PSC* sessions during the first year[median (range)]	11 (1 to 12)	6 (0 to 11)	< 0.001

*PSC: Pre-school consultation ("under-five")

**Figure 1:**
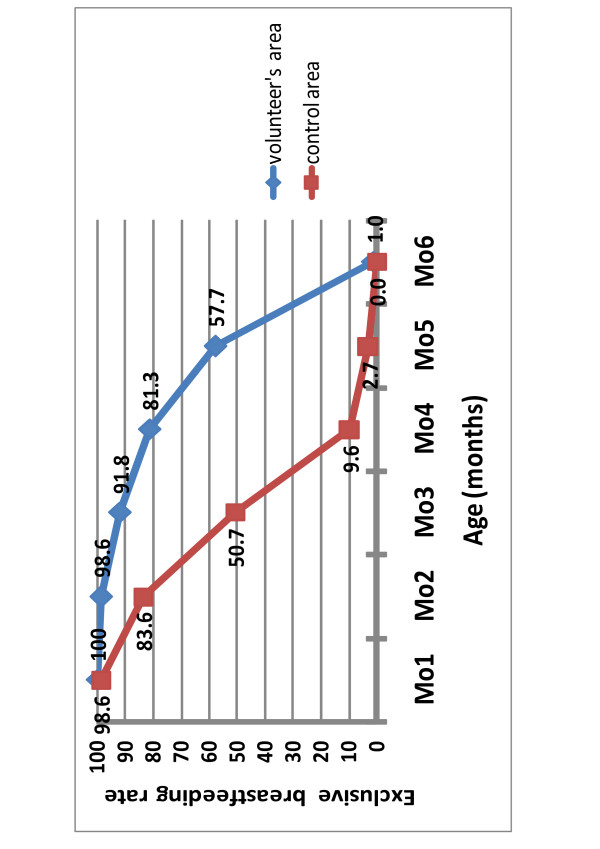
**Proportion of infants receiving exclusive breastfeeding by age for intervention and comparison groups**.

Table 4 summarizes exclusive breastfeeding results in both areas. The median length of EBF since birth was significantly longer in the intervention area than in the comparison area. The proportion of infants who were exclusively breastfed from birth at 4, 5, and 6 months of age was also significantly higher in the intervention area than in the comparison area. Figure 1 clearly shows the difference in the proportion of infants exclusively breastfed from birth starting from two months of age.

**Table 4:** Exclusive breastfeeding

Variable	Intervention area	Comparison area	p value
Median length of exclusive breastfeeding in months (range)	6.0 (2.0 to 7.0)	4.0 (1.0 to 6.0)	< 0.001
Exclusive breastfeeding: % (95% CI)			
4 months age	91.8 (88.1, 95.6)	50.7 (38.9, 62.4)	< 0.001
5 months age	81.3 (75.9, 86.6)	9.6 (2.6, 16.5)	< 0.001
6 months age	57.7 (50.9, 64.5)	2.7 (1.1, 6.6)	< 0.001

CI: confidence interval

The weights of infants at 3, 6, 9 and 12 months in both areas are presented in Table 5. There were no statistically significant differences, but at 12 months the weight of infants in the intervention area tended to be higher than in the comparison area (8.42 kg compared to 7.97 kg; p = 0.055).

**Table 5:** Weight of children in the intervention and comparison groups up to 12 months of age

Age	Group	n	Weight in kg Mean (SD)	p value
**3 months**	Intervention	185	5.42 (0.97)	0.156
	Comparison	95	5.25 (0.82)	
**6 months**	Intervention	16	6.73 (1.23)	0.404
	Comparison	93	6.61 (0.92)	
**9 months**	Intervention	139	7.60 (1.14)	0.176
	Comparison	98	7.41 (1.00)	
**12 months**	Intervention	127	8.42 (1.41)	0.055
	Comparison	33	7.97 (1.02)	

We also looked at the expectations of community volunteers, which are summarized in Table 6. Some suggested that they receive transport fees monthly, uniforms to identify them in the community, free health care, and a salary. Other community volunteers suggested that the district health should recruit them officially into the public health service.

**Table 6:** Expectations of community volunteers

Expectations	n (n = 131)	%
Training	53	41
Salary	36	28
Free health care	16	12
Sharing experiences	14	11
Community projects	12	9

## Discussion

Overall in our study, the community volunteers strategy was highly effective in increasing the rate of EBF from birth until six months of age: At six months, 58% of infants in the intervention group were exclusively breastfed while in the comparison group it was only 3%. Already at four months, a great difference between the two groups could be seen with regard to the continuation of exclusive breastfeeding (92% in intervention group and 51% in the comparison group).

Previous studies carried out in Kivu in 2002 found the rate of EBF at the age of six months to be 20%, the mean of various rural and urban areas [11,12]. But in these studies, the authors had not specified a difference between "exclusive breastfeeding" and "predominant breastfeeding". We consider that the local efforts to raise awareness among pregnant women and actively involving community volunteers helped to improve the rate of EBF.

The key limitation in this study is that we cannot absolutely exclude the start-up effects, which are generally observed in community programs in addition to the effect of observation. This could mean that exclusive breastfeeding was not estimated objectively. However, both the level of education of the community volunteers and their proximity to breastfeeding mothers were a significant advantage for the study. Considering the benefits known to be associated with good breastfeeding practices, involving the community is effective in promoting breastfeeding [6,7,20,21].

Although interventions focused on educating mothers have had mixed effectiveness depending on the studies and the regions, several studies have shown that community projects, which used peer counselors or community volunteers, were able to ensure that breastfeeding was properly promoted. This has been demonstrated recently in three countries in Sub-Saharan Africa: Burkina Faso, Uganda and South Africa [21]. Randomized studies in Bangladesh [22], India [23] and Mexico [24] have also reported similar results in promoting EBF.

Programs based on community involvement have also been associated with a large positive effect on EBF practices in Bolivia and Madagascar [25]. In the three years that a program of community involvement was implemented and supervised by health professionals and community volunteers, introduction of timely initiation of breastfeeding rose respectively from 56 to 74% in Bolivia and from 34 to 78% in Madagascar. On the other hand, as Dyson et al. emphasize, other studies dealing with the promotion of breastfeeding have not shown any effect on the length of time of breastfeeding [13].

In our study, the group of infants from villages, where community volunteers were operating, showed a longer duration of exclusive breastfeeding than that of the control group. This already offers some hope for improving these infants' chances of survival in a context where child mortality is very high.

Our results also suggest that it is important to support breastfeeding women so as to help them continue with exclusive breastfeeding beyond four months. Indeed, in the intervention group, the rate of exclusive breastfeeding remained high until the fourth month and it was from this time onwards that a significant decline was observed. It could be said that for mothers the acceptable period for the introduction of complementary food was not clearly specified (4 or 6 months) or that there were other barriers, which prevented certain mothers from continuing exclusive breastfeeding until six months.

It is possible that the way things were remembered influenced the results one way or another. In practice, this is generally more so in the direction of an overestimation. However, the fact that the prevalence we found in the comparison group was reported also in other inquiries suggests that our observed results were correct. In addition, other inquiries had already indicated the increasing prevalence of exclusive breastfeeding in the health areas with nutritional community programs in the DRC [17].

Bhutta et al. also confirm the promotion of breastfeeding as one part of a range of interventions, which significantly improve survival for children even if its effect on growth remains slight [26]. To our knowledge, there is no community study published, which describes the individual weight gain of individual children in the first year. Kramer et al found that prolonged or exclusive breastfeeding provides no beneficial effects on stature and body mass index [27]. Even if the size of our sample was not appropriate for such an analysis, we did examine the weight gain of infants in our study. Although our data do not show a significant difference between the mean weights in the two groups, there is a slight advantage in the intervention group at 12 months (Table 5). This difference could have been significant if our sample had included more children [28].

As in another Ugandan study [29], we worked with community volunteers, of which the majority were married young women. The volunteers were easily accepted by their community. Although the community volunteers were initially selected as volunteers, they soon demanded material motivation (Table 6).

Regarding the use of health services, this study has shown that community volunteers can improve the utilization of primary health services. Skilled births were very high in the intervention area (93% of newborn compared with 38% in the comparison area). Likewise, mothers and their infants in the intervention area made on average 11 medical visits during their first year compared with 6 medical visits per child in the comparison area. The latter number corresponded to planned immunization sessions for children in the first year. This confirms the problem of the "under-five" clinic system in the DRC, which insures the follow-up of children only during immunization sessions [30].

The improvement of child monitoring during the first year through community volunteers can certainly contribute to reduce child mortality in rural DRC [31,32]. Other authors maintain that community volunteers and community health workers were effective when they were given limited and well defined tasks and were actively supervised by the district health service [33]. We suggest that even in a country that is large and heterogeneous, it is possible to envision community development projects in some areas.

## Conclusion

Our work has shown that promoting breastfeeding by community volunteers improves the duration of exclusive breastfeeding and the use of health services in rural Kivu in the Democratic Republic of Congo. The community volunteers were motivated by the awareness of the seriousness of malnutrition in the region. Other studies with more robust methodology are required to study the operational capacity of community volunteers in the long term and the factors that sustain their motivation.

## Competing interests

The authors declare that they have no competing interests.

## Authors' contributions

GBB and PD produced the original concept for the article and had primary responsibility for the initial draft of the manuscript. All authors contributed substantially to the methods, intellectual content of discussion and finalization of the manuscript.
